# Role of specific IgE to β-lactoglobulin in the gastrointestinal phenotype of cow’s milk allergy

**DOI:** 10.1186/s13223-016-0111-7

**Published:** 2016-02-23

**Authors:** Paloma Poza-Guedes, Yvelise Barrios, Ruperto González-Pérez, Inmaculada Sánchez-Machín, Andres Franco, Víctor Matheu

**Affiliations:** Consulta de Alergia Infantil, Unidad de Alergología-Norte, Hospital del Tórax/Ofra, CHUNSC, Sta. Cruz de Tenerife, 38320 Spain; Immunology, Hospital Universitario de Canarias, La Laguna, Spain; Unidad Alergología-Norte, Hospital Tórax, CHUNSC, Sta Cruz de Tenerife, Spain

**Keywords:** Cow’s milk allergy, Food allergy, Gastrointestinal allergy, Specific IgE, β-lactoglobulin, Casein, Anaphylaxis, IgE-mediated allergy

## Abstract

**Rationale:**

The prevalence of many phenotypes of food allergy is increasing. Specific gastrointestinal (GI) phenotype of food allergy (GI allergy) is also increasing but it is difficult to know the prevalence because of many entities.

**Methods and Results:**

A 1 year retrospective study of pediatric patients complaining exclusively gastrointestinal symptoms after cow’s milk consumption and at least one positive specific IgE (sIgE) to cow’s milk (CM) proteins (CMP) was done (n = 39). The most prevalent symptom was abdominal cramps in 35 patients (90 %), discomfort or abdominal distention in 30 patients (75 %), diarrhea in 10 patients (25 %) and constipation in 5 patients (12 %). IgA anti-transglutaminase antibodies were absent and lactose intolerance was ruled out in all patients. Average of total IgE on this group was 288 UI/ml. sIgE against β-lactoglobulin was the dominant with an average of 4.14 kU/l. sIgE to casein (CAS), which is the dominant protein in systemic anaphylaxis was 1.74 kU/l; sIgE to α-lactoalbumin, the other whey protein, was 0.83 kU/l and sIgE levels to CM were 0.78 kU/l. The quotient sIgE CAS/sIgE β-lactoglobulin in these patients was always lower than 1. Patients experienced an improvement of their symptoms after a CM free diet. An open oral challenge with CM did mimic their initial symptoms in all patients. However, the open oral challenge with dairy products was well tolerated.

**Conclusions:**

Patients with a specific phenotype of GI allergy with CM have specific IgE against β-lactoglobulin, as a dominant sIgE. These patients could beneficiate of a diet with dairy products.

## Findings

Food allergy is currently recognized as a rising problem worldwide. Specific gastrointestinal (GI) food allergy (GI allergy) is also increasing [1] although it is difficult to know the real prevalence because some entities may overlap and many unclear mechanisms are probably involved [1–3]. GI Allergy could be classified into three types according to the implication of immunoglobulin E (IgE) in their pathogenesis, i.e. classical “IgE-mediated entity”; a “combined IgE- and cell-mediated” type and finally, a “cell-mediated/non-IgE-mediated”, consistent with several conditions such as food intolerance or food protein-induced enterocolitis syndrome. Interestingly, those patients afflicted with the GI allergy phenotype develop selective gastrointestinal symptoms exclusively—in contrast to systemic anaphylaxis—hours after the ingestion of offending foods [1, 4]; they probably belong to the cell-mediated/non-IgE-mediated or combined IgE- and cell-mediated food allergy types which are generally diagnosed on the basis of the clinical manifestations [1, 4, 5]. The final pathogenic mechanisms of GI allergy have not been elucidated yet since the diagnostic approaches are not clear and no consensus has been reached either.

### Methods

We performed a 1-year observational retrospective study of patients with selective GI symptoms—i.e. discomfort, abdominal distention, abdominal cramps, flatulence, nausea, vomiting, constipation or intermittent diarrhea only over 30 min after the intake of a glass of cow’s milk. The inclusion criteria also comprise a positive (>0.1 kU/l) serum specific IgE (sIgE) to cow’s milk proteins (CMP) such as casein (CAS), and the main whey proteins such as α-lactoalbumin (ALA) and β-lactoglobulin (BLG). Those patients with confirmed extra-intestinal-cutaneous, ocular, respiratory and/or cardiovascular-symptoms immediately (less than 30 min) occurring after a glass of cow’s milk (CM) were excluded of the study group. Patients with food protein-induced enterocolitis syndrome (FPIES) were also excluded A diagnosis of celiac disease by and a diagnosis of lactose intolerance were also considered as exclusion criteria. Two different groups of subjects were used as control groups, both with markedly different clinical features (a healthy control group without GI CMP reactions and a CMP anaphylaxis) were compared to the study group.

Skin prick tests (SPT) with commercial extracts of CMP were performed (IPI; Stallergenes, Spain). Measurement of the total concentration of IgE (tIgE) in each serum was obtained by enzime-immunoassay (ImmunoCAP, Phadia AB, Uppsala, Sweden). Specific IgE (sIgE) against whole CM, CAS, ALA and BLG were also measured by the (ImmunoCAP) with a detection limit of 0.1 kIU/L. All patients experienced an open challenge test with cow’s milk. Celiac disease and lactose intolerance was ruled out by absence of IgA anti-transglutaminase antibodies [6] by enzyme-linked immunoassay (EliA Celikey IgA, Phadia AB, Uppsala, Sweden) and by hydrogen breath test, respectively. The protocol was approved by the Regional Ethics Committee. Statistical analysis considered significant differences when p ≤ 0.05 (Mann–Whitney U test and Kruskall–Wallis test).

### Results

A total of 336 subjects referring food reactions, out of 1344 pediatric patients were seen in the Outpatient Office during the 12-months study period. Thirty nine subjects (11 %) referred gastrointestinal symptoms exclusively -no other complain at least 30 min after CM consumption- and showed at least a positive sIgE to CMP. Median of age was 5 years–o and 55 % were girls. The most prevalent symptom was abdominal cramps in 35 patients (90 %), followed by discomfort or abdominal distention in 30 patients (75 %), diarrhea in 10 patients (25 %) and constipation in 5 patients (12 %).

SPT with commercial extracts to CMP were positive in only 40 % (16 patients). IgA anti-transglutaminase antibodies were absent in all patients [6]. Average of tgE on this group was 288 UI/ml (median 144 UI/ml), while sIgE against BLG was dominant among the CMP sIgE profile with an average of 4.14 kU/l (median 1.67 kU/l; range 0,21–33) (Table 1). The other two proteins detected were casein, which is the dominant protein in systemic anaphylaxis [7] with an average of 1.74 kU/l (median 0.18 kU/l) and another whey protein, ALA, with average of 0.83 kU/l (median 0.23 kU/l). Average of sIgE levels to CM were 0.78 kU/l (median 0.27 kU/l). The quotient sIgE casein/sIgE BLG in this selected population was always lower than 1 (average 0.3). The healthy control group without GI CMP reactions had levels of sIgE <0.1 kU/l with every single CM protein.

All patients experienced a clinical improvement of their symptoms after the implementation of CM free diet. The clinical improvement was defined as that gastrointestinal discomfort referred in history was completely yield. A new open challenge with a glass of CM reproduced the initial symptoms in all patients. A subsequent open oral challenge with dairy products (one yogurt) was well tolerated in all patients. Additionally these patients maintained taking dairy products on a daily basis diet (fermented such as yogurt or cheese, and baked such as custard).

### Discussion

Bovine BLG, the major whey protein of cow’s milk (~3 g/l), is a small protein, with an 18.4 kDa molecular weight. Unlike the other main whey protein, no clear function has been identified to β-lactalbumin, although a role in the transport of some hydrophobic molecules [8] has been suggested. Among bovine whey proteins, BLG is also a known prevalent allergen, as listed in Annex IIIa of Directive 2000/13/EC and demonstrated by both SPT and oral challenges [9].

The patients with the phenotype of GI allergy have shown low, but positive levels of sIgE against BLG. Patients with this specific phenotype are not susceptible to follow regular CMP desensitization protocols [7, 10], which it has been a hot topic in recent years. The current CMP desensitization protocols have been specifically designed for those patients with the classical anaphylaxis phenotype of IgE-mediated reactions [7], although no guidelines have been developed to determine whose patients may benefit. Interestingly, the quotient sIgE CAS/sIgE BLG in the patients with the GI allergy phenotype was always lower than 1 (average 0.3) compared to those subjects with anaphylaxis, with a quotient higher than 1 [7], given that their dominant allergenic CP is casein (Table 1).

**Table 1:** Figures of levels of specific IgE against some cow’s milk protein fractions in the G-I group and Anaphylaxis group

	GI allergy n = 39			Anaphylaxis n = 20			Significance
	Average	Range	Median	Average	Range	Median	p
Total IgE (IU/ml)	288	16–2360	144	457	295–2500	360	<0.01
Allergen specific IgE (kU/l)							
Whole cow’s milk	0.74	0–2.48	0.26	189	30–586	146	<0.01
Casein	1.74	0–4.8	0.18	178	26–544	90	<0.01
α-lactoalbumin	0.83	0–3.7	0.23	21	9–34	21	<0.01
β-lactoglobulin	4.14	0.10–33.4	1.67	16.2	2.1–38	7.25	<0.01
Ratio casein/β-lactoglobulin	0.30	0.08–0.99	0.22	20	2.1–99	9.5	<0.01

Remarkably 10 patients had levels of sIgE against CM lower than 0.1 kU/l and additionally 12 more subjects showed levels between 0.1 and 0.35 kU/l, which has been the traditional cutoff of many immunoassays of specific IgE. This data is interesting, since many of these patients, with sIgE to CM lower than 0.35 kU/l may be under-diagnosed in the past. The current limit of detection, as low as 0.1 kU/l, for sIgE might help to discriminate among different clinical profiles [11] of allergy. Similarly, ten patients with levels of sIgE against CM lower than 0.1 kU/l, had levels of sIgE to BLG higher than 0.1 kU/l. With these findings, we believe that the determination of several proteins including CAS, ALA and BLG, in addition to specific IgE to CM, should be mandatory. The new detection limits of 0.1 kU/l should always be also recommended. It was only noticed that in these subjects with this phenotype of patients, the sensitivity of sIgE quite higher than the SPT with commercial CM extracts, detecting only 40 % of patients with a positive SPT.

A diet including dairy products but free of CM has been useful with strong positive consequences in their nutrition. Dairy products are those obtained by fermentation of cow milk as yogurt. Just as it has been shown that heat diminishes the reactivity of milk [12, 13] or egg [14], fermentation some dairy products may help to decrease some proteins. In the yogurts it has been shown BLG detection greatly diminished compared to fresh milk measured by chromatography [15]. Even many yogurts have shown an absence of BLG; while other proteins such as CAS and ALA remain present in these yogurts. It is not known the cause why this happens. This could be argued because processing of dairy products could stimulate polymerization of BLG, which is the major whey protein of cow’s milk, in tetramers that probably changes spatial distribution of allergen epitopes [12] and decreasing allergenicity in children and adults with an IgE-mediated cow’s milk allergy [16]. Since there are differences between different products [12], it would also be necessary that manufacturers prove the presence or absence of BLG in natural form to unsure their labeling convinces the requirements to identify BLG in food products. Further studies should be performed to delineate this GI allergy phenotype and if it is suitable to maintain dairy products in these patients. More studies are necessary in defining all possible different milk allergy phenotypes.

## Abbreviations

IgE: immunoglobulin E
GI allergy: gastrointestinal food allergy
sIgE: specific IgE
tIgE: total IgE
CM: whole cow’s milk
CMP: cow’s milk proteins
CAS: casein
ALA: α-lactoalbumin
BLG: β-lactoglobulin
SPT: skin prick tests
